# Radical surgery for anal canal neuroendocrine carcinoma with pagetoid spread: a case report

**DOI:** 10.1093/jscr/rjab111

**Published:** 2021-05-18

**Authors:** Sotaro Fukuhara, Masanori Yoshimitsu, Takuya Yano, Ko Oshita, Kensuke Bekku, Hitoshi Okamoto, Yoichiro Toi, Koichi Ichimura, Wataru Okamoto, Masazumi Okajima

**Affiliations:** Department of Surgery, Hiroshima City Hiroshima Citizens Hospital, 7-33, Motomachi, Naka-ku, Hiroshima 730-8518, Japan; Department of Surgery, Hiroshima City Hiroshima Citizens Hospital, 7-33, Motomachi, Naka-ku, Hiroshima 730-8518, Japan; Department of Surgery, Hiroshima City Hiroshima Citizens Hospital, 7-33, Motomachi, Naka-ku, Hiroshima 730-8518, Japan; Department of Surgery, Hiroshima City Hiroshima Citizens Hospital, 7-33, Motomachi, Naka-ku, Hiroshima 730-8518, Japan; Department of Urology, Hiroshima City Hiroshima Citizens Hospital, 7-33, Motomachi, Naka-ku, Hiroshima 730-8518 Japan; Department of Plastic Surgery, Hiroshima City Hiroshima Citizens Hospital, 7-33, Motomachi, Naka-ku, Hiroshima 730-8518, Japan; Department of Dermatology, Hiroshima City Hiroshima Citizens Hospital, 7-33, Motomachi, Naka-ku, Hiroshima 730-8518, Japan; Department of Pathology, Hiroshima City Hiroshima Citizens Hospital, 7-33, Motomachi, Naka-ku, Hiroshima 730-8518, Japan; Cancer Treatment Center, Hiroshima University Hospital, 1-2-3 Kasumi, Minami-ku, Hiroshima 734-8551, Japan; Department of Surgery, Hiroshima City Hiroshima Citizens Hospital, 7-33, Motomachi, Naka-ku, Hiroshima 730-8518, Japan

## Abstract

Anal canal neuroendocrine carcinoma (NEC) with pagetoid spread (PS) is a rare disease, and its treatment strategy remains unclear. The prognosis of anal canal NEC with PS is poor. Resection margin status is very important for anorectal carcinoma because it affects survival. When accompanied by PS, the defect of the resulting perineal wound following radical surgical intervention may be necessarily enlarged to ensure the appropriate margin status. This case report discusses the treatment of a patient with advanced anal canal NEC with PS, inguinal lymph node metastasis and sphincter infiltration in which total pelvic exenteration with plastic surgery was successfully performed. The plastic surgery incorporated a gracilis muscle flap that was useful for the reconstruction of the enlarged perineal defect.

## INTRODUCTION

Neuroendocrine carcinoma (NEC) is an extremely rare form of anal cancer. Anal canal NEC is often associated with distant metastases, and prognosis is poor [1]. Due to its rarity, the treatment strategy for anal canal NEC remains unclear. Pagetoid spread (PS) is included in secondary perineal Paget disease, which is one of the extramammary Paget diseases [2]. Herein, we present a case report for the treatment of advanced NEC with PS, inguinal lymph node metastasis and sphincter infiltration.

**Figure 1:**
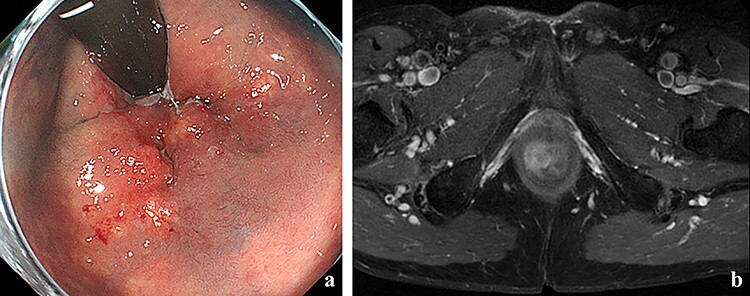
(**a**) Colonoscopy showed erythema of the mucous membrane from the anal canal to the rectum; (**b**) MRI showed that the levator ani muscle was ruptured by the tumor.

**Figure 2:**
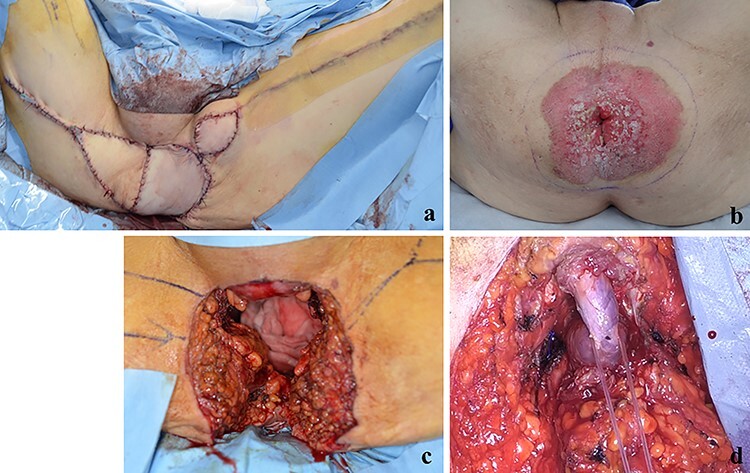
(**a**) The perineal defect was reconstructed using the gracilis muscle flap; (**b**, **c**) PS lesion was excised with a margin of 1 cm or more; (**d**) the bulbar urethra was exposed from the perineum.

## CASE PRESENTATION

**Figure 3:**
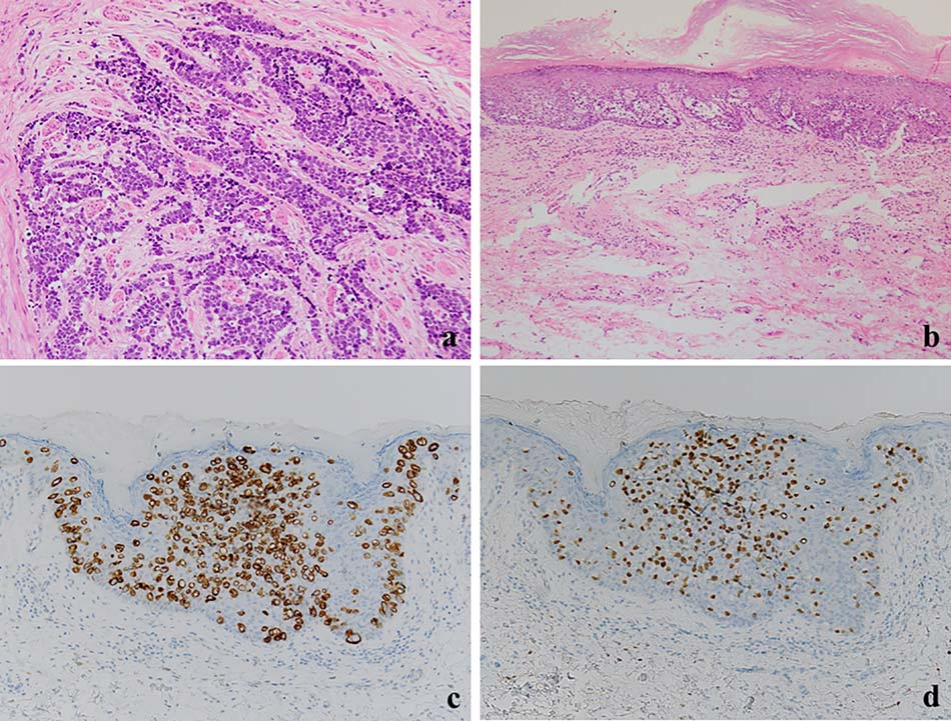
(**a**) The tumor cells with a high nuclear cytoplasmic ratio nuclei proliferated with compact nests in the anal canal (×100); (**b**) the tumor cells did not show neuroendocrine differentiation and were only components of tubular adenocarcinoma in the perianal lesions (×200); (**c**, **d**) The tumor cells were positive for CK20 and CDX2, respectively (×100).

A 70-year-old man who complained of refractory perianal skin ulcers was referred to our hospital. The condition had spread concentrically around the anus. Colonoscopy revealed erythema of the mucous membrane that was continuous from the anal canal to the rectum (Fig. 1a). Magnetic resonance imaging (MRI) revealed a hyperintense mass in the right wall of the anal canal on T2-weighted images. Tumor invasion of the prostate and levator ani muscle was suspected (Fig. 1b). Enhanced computed tomography (CT) showed a 3-cm wall thickening in the right wall of the anal canal and swelling of the left inguinal lymph node. Fluorine-18 fluorodeoxyglucose positron emission tomography CT (FDG-PET/CT) showed FDG uptake in the anal canal with a maximum standardized uptake value (SUVmax) of 5.4. The left inguinal lymph node had a SUVmax of 2.5. Biopsy was performed on the anal canal and perianal skin lesions. The pathological results indicated adenocarcinoma with severe atypia. Immunohistochemically, the tumor of the perianal skin lesion was positive for cytokeratin 20 (CK20) and caudal homebox transcription factor 2 (CDX2). Based on the above results, the tumor was diagnosed as anal canal adenocarcinoma with PS, inguinal lymph node metastasis and infiltration into the prostate and sphincter. It was decided that radical resection was possible and total pelvic exenteration (TPE) was performed with perianal plastic reconstruction using the gracilis muscle flap and D3 lymphadenectomy (Fig. 2a). The perianal skin lesion was excised with a margin of 1 cm or more (Fig. 2b and c), and simple closure was challenging. The bulbar urethra was pulled out from the perineum to secure a urethral stump (Fig. 2d). The pathological results revealed that the surgical margin was negative. The tumor cells of the anal canal had nuclei with a high nuclear cytoplasmic ratio and infiltrative proliferation with compact nests (Fig. 3a) and were positive for CD56, chromogranin A and synaptophysin. The majority were NEC, and only a few were tubular adenocarcinoma. The Ki-67 labeling index was greater than 70%. The tumor cells of the perianal skin lesions were adenocarcinoma and had no NEC features and were negative for chromogranin A and synaptophysin and positive for CK20 and CDX2 (Fig. 3b–d). In addition, sphincter infiltration and inguinal lymph node metastasis were observed. Accordingly, a diagnosis of anal canal NEC with PS, inguinal lymph node metastasis and sphincter infiltration was made. Although debridement and local flap was required 17 days after the operation because necrosis was observed in a part of the flap, the course after that was generally good. The patient was discharged 42 days postoperatively. At the 7-month follow-up after resection, the patient did not show any recurrence.

## DISCUSSION

NEC is present in only approximately 1% of anal canal malignant tumors [3]. To the best of our knowledge, there have been very few reports of PS associated with anal canal carcinoma and only one report of anal canal NEC [4]. Therefore, treatment strategies for anal canal NEC remain unclear due to its rarity, especially for those with PS.

Aytac *et al*. reported on the prognosis and treatment of 25 cases of NEC of the colon, rectal and anal canal. Chemotherapy or chemoradiotherapy (CRT) was performed in cases with distant metastasis, while surgery alone or surgery with chemotherapy or neoadjuvant chemoradiotherapy (NCRT) was performed in cases with no distant metastasis. This study confirmed the poor prognosis of these rare tumors [5]. The resection margin status is very important in the surgical treatment of anorectal carcinoma. For advanced rectal cancer, the resection margin affects survival, and pelvic exenteration can improve the resection margin status [6]. Extramammary Paget’s disease has been reported to require a 1-cm resection margin to make the margins negative [7]. Given these requirements, excision of PS lesions with sufficient margins may substantially widen the perineal defect. There were cases in which the perineal defect was left open [8]. Management of an open perineal defect takes a long time to heal, resulting in poor quality of life (QOL). In our case, the perineal healing period was shortened by reconstructive plastic surgery and sufficiently secured resection margins. In addition, due to the preoperative suspicion of infiltration of the prostate and the levator ani muscle, it was also considered important to ensure sufficient margin status of the urethra. Exposing the bulbar urethra from the perineum approach allowed dissection with a good visual field for securing the resection margin of the urethral.

It has been hypothesized that NEC could originate from the differentiation of pluripotent stem cells, preceding adenocarcinoma or the mechanical stimulation [9]. In previous reports of anal canal NEC or adenocarcinoma with neuroendocrine features accompanying PS, the tumor cells of PS had NEC features [4, 10]. In our case, the tumor cells of the perianal lesions had no NEC features. It is possible that anal canal NEC and primary extramammary Paget’s disease were present at the same time. However, it is reasonable to regard them as a series of conditions in which anal canal NEC exhibited PS because the perianal lesions were positive for CK20 and CDX2. Thus, NEC in our case is likely to be derived from the preceding adenocarcinoma and therefore had common origin.

In conclusion, we succeeded in performing radical surgery using TPE with plastic reconstruction for advanced NEC with PS. Reconstructive plastic surgery using the gracilis muscle flap also proved useful for treating the enlarged perineal defect. This is also an important case for helping clarify the origin of the NEC.
